# DNA Methylation in Genetic and Sporadic Forms of Neurodegeneration: Lessons from Alzheimer’s, Related Tauopathies and Genetic Tauopathies

**DOI:** 10.3390/cells10113064

**Published:** 2021-11-07

**Authors:** Geraldine Zimmer-Bensch, Hans Zempel

**Affiliations:** 1 Functional Epigenetics in the Animal Model, Institute for Biology II, RWTH Aachen University, 52074 Aachen, Germany; 2 Research Training Group 2416 MultiSenses-MultiScales, Institute for Biology II, RWTH Aachen University, 52074 Aachen, Germany; 3 Institute of Human Genetics, Faculty of Medicine and University Hospital Cologne, University of Cologne, 50931 Cologne, Germany; 4 Center for Molecular Medicine Cologne (CMMC), Faculty of Medicine and University Hospital Cologne, University of Cologne, 50931 Cologne, Germany

**Keywords:** Alzheimer, tauopathy, TAU, *MAPT*, epigenetics, neurodegeneration, neurogenetic disease, DNA methylation

## Abstract

Genetic and sporadic forms of tauopathies, the most prevalent of which is Alzheimer’s Disease, are a scourge of the aging society, and in the case of genetic forms, can also affect children and young adults. All tauopathies share ectopic expression, mislocalization, or aggregation of the microtubule associated protein TAU, encoded by the *MAPT* gene. As TAU is a neuronal protein widely expressed in the CNS, the overwhelming majority of tauopathies are neurological disorders. They are characterized by cognitive dysfunction often leading to dementia, and are frequently accompanied by movement abnormalities such as parkinsonism. Tauopathies can lead to severe neurological deficits and premature death. For some tauopathies there is a clear genetic cause and/or an epigenetic contribution. However, for several others the disease etiology is unclear, with few tauopathies being environmentally triggered. Here, we review current knowledge of tauopathies listing known genetic and important sporadic forms of these disease. Further, we discuss how DNA methylation as a major epigenetic mechanism emerges to be involved in the disease pathophysiology of Alzheimer’s, and related genetic and non-genetic tauopathies. Finally, we debate the application of epigenetic signatures in peripheral blood samples as diagnostic tools and usages of epigenetic therapy strategies for these diseases.

## 1. Introduction

### 1.1. Alzheimer’s Disease and Other Tauopathies

Neurodegenerative Diseases (NDDs) involve the irreversible loss of neurons or neuronal functions. The term NDD is often used to describe conditions of the central nervous system (CNS) characterized by neuronal dysfunction, neuronal loss and brain atrophy. The overwhelming majority of NDD-patients suffer from Alzheimer’s Disease (AD), the most common form of tauopathy. Tauopathies are a heterogenous group of diseases, all characterized by abnormal accumulations and aggregations of the neuronal protein TAU [1,2,3].

TAU is a microtubule-associated protein (MAP) widely expressed in neuronal tissues and diverse cell types, in particular neurons and oligodendrocytes, but usually not in astrocytes. Roughly 80% of patients suffering from dementia are affected by a tauopathy [4]. In the clinic, patients are diagnosed with cognitive/dementia syndromes, movement disorders, motor neuron disease, either with isolated disease manifestation or in various assemblage [5], caused by the loss of neuronal function of the brain parts affected by TAU pathology. There is no such thing as a clinically well-defined single entity “tauopathy”. Tauopathies can be classified as (i) primary tauopathies, with TAU being either the predominant or the causative pathology, and (ii) secondary tauopathies, where TAU pathology is either secondary to or appears in combination with other brain pathologies or insults (see below). Other classifications are e.g., the syndromic classification by Höglinger et al., that separates cognitive syndromes from motor syndromes (see e.g., [5]). Primary tauopathies may show an aggregation or pathology only of 3-repeat (3R) or 4-repeat (4R) TAU isoforms, while most secondary tauopathies present with an aggregation of all isoforms (3R+4R/mixed). Hence, tauopathies can also be classified into 3R, 4R or mixed tauopathies, and can be based on structural features (i.e., the filament folds) of the aggregates [6,7,8]. As the same genetic mutation in the case of genetic forms of tauopathies [9,10], or different secondary tauopathies [11,12,13,14] can display similar or very different disease manifestations or molecular changes of TAU, we here roughly reclassify tauopathies as either clearly genetic or epigenetic, idiopathic/sporadic, secondary/existing copathology, and (likely) environmental (for a list of tauopathies see Table 1).

AD and closely related tauopathies (e.g., Frontotemporal Dementia (FTD) variants), share clinical features, such as age of onset and several symptoms impeding clinical differentiation. Yet cause, histopathology and pathomechanism, disease onset and progression, as well as many symptoms (see below and Table 1), differ with respect to the subform of tauopathy, which needs to be taken into consideration when referring to tauopathy and related therapies. Because of its tragic popularity, AD is the best investigated and defined disease entity in the canon of tauopathies, for which we will briefly discuss AD in more detail.

#### Clinics of AD

Clinically AD is characterized by cognitive dysfunction ultimately resulting in dementia. The term dementia describes the loss of once acquired cognitive functions, independent of age-associated decline in cognitive abilities. Cognitive impairment (CI) in AD typically begins with subtle failure of memory that may not be recognized clinically, but may be perceived by the affected patient [15]. This very subtle CI may develop into “mild cognitive impairment” (MCI) stage, with detectable failure of memory when rigorously tested. Not all patients affected by MCI progress to AD: Progression of MCI to full blown dementia occurs with a 60% chance over ten years. Progression of MCI to AD can be predicted in experimental settings either alone or by combining clinical, imaging-based, and laboratory diagnostics [15,16]. Social behavior changes noted first by relatives and care-takers include confusion, poor judgment, language disturbance, visual complaints, agitation, withdrawal, and hallucinations. Less frequently, but complicating discrimination from other dementia syndromes, Parkinson-like motor phenotypical particularities, augmented muscle tone, a broad spectrum of seizures & myoclonus, incontinence, and mutism become clinically detectable and also apparent for relatives and care-takers. Patients usually succumb to accompanying inanition but also simply due to malnutrition, pneumonia or other typical diseases of bed-ridden patients [17,18]. Approximately >95% of all AD is late onset (LOAD; age > 60–65 years) and about <5% is early onset (EOAD; age < 60–65 years). The risk of Alzheimer’s is 60–80% dependent on heritable factors (clinical aspects extensively reviewed elsewhere [17,19,20]).

### 1.2. Pathomechanisms of AD—A Role of Aβ and TAU in Disease Progression (Neuroanatomics, Clinics and Imaging Level)

In AD, extracellular deposition of plaques composed of the Amyloid-beta (Aβ) protein, and intracellular aggregation of the protein TAU, are pathognomonic of the disease. Post-mortem, AD patient brains display diffuse extracellular Aβ plaque, neurofibrillary tangles (NFTs), which are localized intracellularly (except for “ghost-tangles”, remnants of cells that succumbed to TAU pathology), and neuropil threads of hyperphosphorylated TAU, alongside marked neurodegeneration [20]. Aβ and TAU, in the case of AD both have to present in aggregated states for the histopathologic diagnosis of AD, are seen as triggers and executors of disease cause and progression, with Aβ accumulation usually placed upstream in the pathological cascade [1,2,20,21,22]. The local or global decrease in glucose consumption, observable at late stages, is in turn considered a downstream consequence [23]. Aberrant production of Aβ, a 37–43 amino acid long peptide cleaved out of the Amyloid-Precursor-Protein (APP) by inter alias (i.a.) a complex composed of PSEN1/PSEN2, and Aβ deposition in the form of amyloid plaques occur 20–30 years before the onset of detectable CI. Aberrant production (and changed ratios) of Aβ is commonly placed as the upstream event/initial trigger of the disease. However, the “amyloid hypothesis” has been debated for more than 30 years [24,25,26]. In genetic forms of AD manifesting as EOAD, the overwhelming known disease-causing mutations (namely *APP, PSEN1, PSEN2,* see below and Table 1) lead to an increase or aberration of Aβ production. Yet, in mouse and cell culture models of AD, exposure or overproduction of Aβ is without effect if the TAU protein is knocked-out (or otherwise suppressed): This clearly hints towards TAU and TAU misdistribution as an essential executor in mediating neurodegeneration and cognitive dysfunction [2,27,28,29].

Apart from aberrant Aβ-production, the deposition of Aβ may be an upstream event in AD, but does not correlate with CI. The histopathological hallmark of AD that correlates best with clinical symptoms and synapse loss, in particular with CI, is the progressive accumulation and aggregation of TAU. Disease progression from MCI to AD and structural/functional brain deterioration is also more closely related to TAU aggregation, and possibly may be predicted by TAU (PET-) imaging [30,31,32]. In addition, the appearance of TAU in specific cognitive networks leads to brain region/domain-specific cognitive impairments that can be explained by the loss of physiological function of the affected brain region, indicating a clear causative role for TAU accumulation and aggregation for impairing brain function [33]. On a population basis, a stereotypical progression derived from histopathological stainings at autopsy, formalized via the Braak staging system, exhibits a cortical spreading of TAU aggregates: The first appearance of TAU tangles is in the transentorhinal cortex. Tangles subsequently spread throughout the medial and basal temporal lobes, then into neocortical associative regions, and finally into the unimodal sensory and motor cortex [34,35]. This spreading pattern is observable on an averaged population basis. However, advances in PET-imaging have improved output from longitudinal clinical studies. Artificial intelligence (AI)-based data interpretation has very recently allowed to better correlate the clinical symptoms in AD with structural and TAU-based brain abnormalities, revealing four different subtypes of AD, which can now be described clinically and in close correlation using TAU-based PET-imaging [36]. In sum, Aβ deposition may be an upstream trigger or indicator of disease, but from a clinical, anatomical and imaging-based perspective aberrant deposition and other changes of TAU must be the main driver of AD pathology and consequent CI.

### 1.3. The Role of TAU in Neuronal Dysfunction on a Cellular & Molecular Level

What are the molecular and cellular functions of TAU, and how are they impaired in disease? The TAU protein is a microtubule-associated protein (MAP), and can bind to and promote the assembly of microtubules. TAU expression is up-regulated during neuronal differentiation together with tubulin [37]. In mature CNS neurons, TAU is present in neuronal axons and not evident in dendrites, and several mechanisms have been proposed for the axonal targeting of TAU (i.a. RNA- and protein-based, for review see [2]). Human TAU is encoded on chromosome 17q21 [38], and the human CNS comprises six major (alternatively spliced) isoforms, with different isoform ratios depending on the developmental stage, cell type, and brain region [39,40]. A balanced isoform expression and epigenetic regulation of the isoforms seem to be crucial for both sporadic and genetic TAU-related diseases: Mutations in intronic *MAPT* regions that lead to an altered isoform expression of TAU, but not the overall protein amount or the amino acid sequence, are sufficient to cause Frontotemporal Dementia with tauopathy (FTD-TAU) reminiscent of AD and Progressive Supranuclear Palsy (PSP) [41,42]. Further, proper intracellular axodendritic distribution of TAU, i.e., successful axonal targeting, may be essential to avoid neuronal dysfunction. In virtually all tauopathies, the TAU protein is mislocalized and/or ectopically expressed, with mislocalization of the TAU protein (“TAU missorting”) being the earliest sign of disease progression (for review see [1]). The individual TAU isoforms display differential sorting and expression patterns [43,44]. TAU missorting to dendrites and spines, a very early sign of the disease, is associated with synaptic dysfunction, loss of microtubules and mitochondria, which likewise represent early signs of neurodegeneration in AD. Clearly, elucidating the molecular and cellular regulation of TAU is crucial to unravel the pathomechanism of AD and related tauopathies. The epigenetic mechanisms regulating intracellular and splice-isoform distribution are unresolved, but may be essential to elucidate key components of TAU driven-toxicity in AD and related tauopathies: Sorting mechanisms are in part RNA-based, and epigenetic regulation of splicing is likely [2].

The role of TAU as a driver of neuronal dysfunction becomes even clearer when we look beyond AD: The aberrant deposition of TAU (mainly in the somatodendritic compartment) is a common feature of tauopathies [1]. The formation of neurofibrillary tangles (NFTs), neuropil threads including hyperphosphorylated TAU protein, is the histopathological hallmark of most tauopathies. This holds true not only for AD, but also for Frontotemporal Dementia (FTD) and variants thereof, such as Pick’s Disease (PiD), Corticobasal Degeneration (CBD), Progressive Supranuclear Palsy (PSP), as well as other common (e.g., Parkinson Disease (PD) Traumatic Brain Injury (TBI), both of which are clearly secondary to alpha-synuclein deposition and physical injury, respectively) and rare genetic diseases [45,46,47,48] (see also Table 1 for an exemplary list of genetic, epigenetic, primary, and putative secondary tauopathies).

### 1.4. Established Genetic Mechanisms of AD and Tauopathies

For autosomal-dominant inheritable forms of early onset AD, mutations in the genes *APP, PSEN1,* and *PSEN2* coding for the amyloid-precursor-protein, presenelin-1, and presenelin-2, respectively, are causative for the disease. Homozygote carriers of the ε4 allele of the apolipoprotein E, encoded by *APOE4*, have an ~15-fold increased risk to be affected by EOAD or LOAD. Heterozygote carriers still have a 3-fold higher risk, but the overall risk is below 35% and 20%, respectively to be affected by AD, while the ε2 allele is clearly protective. The ε3 allele is considered baseline/background population. In the last 10 years, mainly GWAS but also genome studies have revealed more than 20 (and possibly up to 75 according to recent preprints) risk loci/genes that modulate disease risk. While the odds ratio for *APOE2* and *APOE4* for disease risk ranges from 0.56 to 14.49, the odds ratio for other genes lies between 0.68 (*PLCG2*) and 2.08 (*CD2AP*). Of note, loci in the vicinity of *MAPT* are associated with a reduced odds ratio for AD (0.73 or 0.94), indicating that *MAPT* can also modify the risk for AD (for a full list of current risk genes and odds ratios we refer to other review articles [49,50,51].

Genetic forms of AD and related tauopathies, as well as the histopathology and imaging-based findings have elucidated possible connections and allowed initial mapping of cellular components and pathways involved. Still, genetic and signaling-based mechanisms alone can by far not exhaustively describe the pathomechanisms both of genetic and sporadic forms of neurodegeneration, in particular of AD and related pathology. Epigenetic mechanisms likely bridge the gaps in current knowledge of tauopathy disease mechanisms, and might provide a mechanistic basis for certainly existing environmental triggers.

## 2. Implication of DNA Methylation in AD and Tauopathies

Together with histone variants and modifications, alterations in nucleosome positioning, non-coding RNAs, and DNA methylation constitute the epigenetic toolkit. As enormous progress was made in investigating the functional implications of DNA methylation in the context of AD and tauopathies, we will mainly focus here on the discussion of this epigenetic modification. For more detailed information on the role of histone modifications in AD and tauopathies, we refer to other excellent reviews [58,59].

DNA methylation describes the chemical modification of the DNA itself by the addition of methyl groups mostly on cytosines, but also on adenines via DNA methyltransferases (DNMTs) [60], with DNMT1 and DNMT3A being the major DNMTs in the CNS [61]. DNA methylation effects, i.a. transcriptional control when occurring at enhancer and promoter sites, alternative promoter choice and alternative splicing [62,63].

At the level of transcriptional regulation, methylated motifs of transcription factor (TF) binding sites physically impede the binding of methyl-sensitive TFs, leading to transcriptional suppression. Furthermore, the interaction of the methyl-CpG-binding domain proteins (MBDs) with methylated DNA prevents binding of TFs and promotes inactive heterochromatin formation by recruiting other chromatin and nucleosome remodeling factors [64]. Recent studies, however, suggest that DNA methylation marks may also create binding motifs for certain TFs that do not possess a methyl binding domain [65]. In silico studies propose an augmenting number of TFs predicted to bind methylated DNA loci, and certain TFs might even recognize distinct sequences depending on the DNA methylation state [66].

DNA methylation can be dynamically reconfigured involving Ten-eleven translocation (TET) family enzyme-dependent mechanisms that initiate active DNA demethylation also in neurons, in addition to passive DNA demethylation in replicating cells [67]. TET-mediated oxidation of 5-methylcytosine (5mc) to 5-hydroxymethylcytosine (5hmc) and iterative oxidation forms enable the active reversion to cytosine by thymine DNA glycosylase (TDG)-mediated base excision repair [68,69], also in neurons [70].

Epigenetic mechanisms concertedly modulate chromatin structure and gene expression. E.g., particular histone modifications predispose for the set-up of DNA methylation signatures and vice versa [64,71]. Conversely, DNMTs influence histone modifications by transcriptional control of genes coding for enzymes of histone modifying complexes. Further, DNMTs interact e.g., with histone modifying complexes, such as Polycomb Repressor Complex 2 (PRC2) at the protein level [72,73]. Moreover, lncRNAs were reported to be involved in targeting DNMTs and histone modifying complexes to discrete genomic loci [74,75].

### 2.1. Age-Dependent Changes of DNA Methylation Marks and the Relevance for AD and Tauopathies

Genomic instability, aberrant gene expression, and the loss in chromatin structure are features of both aging and multifactorial diseases such as AD [76,77]. These alterations are intimately associated to epigenomic changes [78], and can be responsive to environmental influence [79]. Aging represents the main risk factor for AD and most tauopathies, hence, age-associated epigenetic alterations likely contribute to the structural and functional changes of the brain that lead to progressive cognitive deficits and possibly derived augmented susceptibility to neurodegenerative disorders such as AD and tauopathies [80,81].

A common hallmark of both healthy aging and AD/tauopathies is the decline in memory function. Changes in the gene expression of chromatin remodeling enzymes, such as DNMTs and histone modifying proteins, are associated with alterations in synaptic plasticity, learning and memory [82,83,84,85,86,87]. Moreover, the expression or activity of epigenetic modifiers is altered in the aging brain [88]. Together, this underlines the relevance of epigenetic modifications in the context of aging and AD-related symptoms, which will be discussed as follows.

The age-related decline in *Dnmt3a2* expression seems to be linked to diminished cognitive abilities, as these were restored upon the rescue of decreased *Dnmt3a2* levels in mice [89]. In line with the decline in DNMT expression upon aging, global hypomethylation with local sites of hypermethylation were observed in aging brains across species, affecting the expression of genes related to synapse function, cellular homeostasis but also neuronal development [90,91]. Such age-associated DNA methylation changes are proposed to contribute to transcriptional alterations of AD-related genes, possibly predisposing for the disease [92,93]. Indeed, the expression levels of key genes associated with AD and taupathypathophysiology may be regulated by DNA methylation in an age-dependent fashion. This is true e.g., for the membrane protein APP (Amyloid-Precursor Protein), concentrated in the synapses. As indicated above, mutations in the *APP* lead to EOAD, due to an augmented or aberrant generation of the Aβ protein. The APP coding gene, which is frequently methylated, displays an age-related demethylation of cytosines in the promoter region (those at −207 to approximately −182), suggested to be linked to the Aβ deposition in the aged brain [94,95]. In contrast, the promoter regions of the neprilysin (NEP) gene, known to inhibit AD occurrence by clearing Aβ in the brain, turned out to be highly methylated and down-regulated in AD and aged healthy brains [96,97]. The elevated methylation of the *NEP* gene results in decreased expression, negatively impacting Aβ clearance, possibly causative for the elevated Aβ plaque burden in AD [96].

Also, methylation status of cytosines in the promoter region of the *MAPT* gene changes with age to reduce *MAPT* transcription in the cerebral cortex in humans: While in the binding sites of the transcriptional activator SP1 a significant age-related increase in 5mC was observed on autopsy, a decrease with age of 5mC in the binding sites for GCF, and a repressor of GC-rich promoters was revealed [94].

Global DNA methylation changes in the brain, but also in peripheral tissues including the blood [98,99], have been identified to correlate well with aging. This epigenetic clock has even been used to predict the chronological (actual) age [99], hence serving as a measure of age-acceleration when comparing the biological (estimated) with the chronological age of an individuum. Age-acceleration has been associated with diminished physical and cognitive fitness [100], and an increase in all-cause mortality [101], but also with a range of age-related diseases, such as AD [102]. Due to this, the epigenetic clock is discussed as a biomarker of aging and age-related disorders, such as AD [103,104,105], as well as of disease progression [106].

### 2.2. Evidence for the Implication of Altered DNA Methylation Signatures in AD and Tauopathies

Similar to the aging brain, global DNA hypomethylation was reported for AD, supported by decreased immunoreactivity for 5mc in cortical neurons of postmortem AD brains (hippocampus, entorhinal and prefrontal cortex, cerebellum) compared to controls [105,107,108], in line with diminished staining with antibodies directed against DNA methylation maintenance factors in the hippocampal tissue of AD patients [107]. Monozygotic twin studies collecting twin pairs discordant for AD found reduced levels of DNA methylation in neuronal nuclei of the AD twin in the temporal neocortex [109].

Neuronal and glia cell-type specific differential methylation dynamics associated with AD Braak stage progression were observed for genes such as *ANK1, MCF2L, STK32C, LRRC8B, MAP2* and *S100B*, and methylation changes at the key AD risk genes *APP* and *ADAM17* were identified in a meta-analysis [110]. The increased risk of dementia and AD was further correlated with elevated DNA methylation levels in the promoter region of *APOE* [111]. Genetic variation in the *APOE* gene is related to AD risk and Aβ burden, with the *APOE4* variant being the most consistent (see above) genetic risk factor [112,113]. The DNA methylation-dependent effect was, however, independent of the *APOE* genotype [111]. This points to an independence of allelic and methylation variation of *APOE* for the risk to develop dementia.

#### 2.2.1. DNA Methylation Changes Lead to Pathological Phosphorylation of TAU

Disturbed methylation levels in the promoter regions of genes related to TAU phosphorylation, which plays a critical role in tauopathies, were revealed by diverse clinical and basic research studies in the context of AD [114]. GSK3β is the kinase most commonly implicated in hyperphosphorylation of the TAU protein, which in turn is believed to be a prerequisite for the aggregation and formation of NFTs [115]. During early AD development, low DNA methylation levels were found in the promoter region of the GSK3β gene (*GSK3B)* in the prefrontal cortex tissue of AD patients, and consequently GSK3β expression was increased in patients with initial AD [116]. While at Braak stages I-II, a decrease of the inactive GSK3β was found in the cortex from AD patients, a considerable increase was observed in AD patients at stages V-VI compared to control subjects. The authors propose that GSK3β hyperactivity, and then NFTs formation, could be initiated at an early stage of the disease and turned off at the final stages [116].

TAU hyperphosphorylation is further driven by up-regulated *Cdk5* expression, causing diminished long-term synaptic potentiation and culminating in impairments of spatial learning and memory. Low levels of cytosine methylation were detected in the promoter region of *Cdk5* in the hippocampal CA1 region in a rat model with Aβ-induced memory deficiency [117].

Increased DNA methylation, linearly correlating with the Braak stage, was observed in the promoter region of the dual specificity phosphatase 22 gene (*DUSP22*) in the hippocampi of AD patients. A reduced DUSP22 expression was detected at mRNA and protein levels, and as DUSP22 was found to inhibit PKA-mediated TAU phosphorylation [118], its reduced expression could have direct consequence for disease progression. In addition to TAU, PKA activates the Ser133 phosphorylation of the cAMP response element-binding protein (CREB). CREB is relevant for neuronal function and synaptic plasticity, long-term memory formation and neuronal survival regulation [119], all of which is compromised in AD [18]. Hence, the reduced DUSP22 expression could promote neuronal survival through elevated PKA/CREB activation. The authors propose the increase in the *DUSP22* promoter methylation to be a consequence of Aβ-induced toxicity, in the sense that cells respond with active methylation to improve their survival [118].

TAU phosphorylation can further be influenced by TET-dependent DNA demethylation. BDNF, as a key component in the maintenance of synaptic plasticity and synaptogenesis in the hippocampus [120], is closely related to TAU hyperphosphorylation [121,122]. The BDNF chromatin status and promoter accessibility is regulated by TET1 and ERK1/2 [123], indicating that TET1-dependent *BDNF* DNA demethylation may influence TAU phosphorylation. Overall, these studies provide evidence for an implication of DNA methylation dependent transcriptional control of TAU phosphorylation-related genes in AD. Most of these genes are proposed effectors downstream of Aβ pathology, but upstream of TAU pathology that is more closely related to cognitive dysfunction in patients. Hence, targeting these genes might disrupt the amyloid cascade upstream of TAU, for which these genes represent potential targets for AD treatment strategies.

#### 2.2.2. Altered DNA Methylation Signatures as a Consequence of Disease Pathophysiology, Such as Aβ Burden and TAU-Phosphorylation

Changes in DNA methylation could be caused by the altered neuronal physiology in AD and tauopathies, such as the accumulation of Aβ peptides [118,124]. Hence, altered epigenetic signatures could be a bystander of disease progression, leading to the devastating dysregulation of genes and driving the further progression of neurodegeneration in AD and other tauopathies. Furthermore, distinct mutations associated with these diseases could elicit “secondary” changes in the DNA methylation pattern. It is well-known that changes in DNA sequence trigger alterations in DNA methylation signatures [125,126].

#### 2.2.3. Aβ Peptide and TAU-Phosphorylation-Driven Changes in the Expression and Localization of DNA Repair Related Proteins

Disruption of the maintenance of genomic integrity emerges to play a central role in AD and related tauopathies [127]. Early intraneuronal accumulation of Aβ peptides promotes global DNA hypomethylation and thereby an increased expression of genes involved in DNA repair, i.a. *BRCA1*, in a mouse model of AD [128]. *BRCA1* was up-regulated in response to Aβ stimulation, in both cellular in vitro and in vivo mouse models, acting neuroprotectively against Aβ-induced DNA double-strand breaks. Up-regulated expression of *BRCA1* was further observed in postmortem brain samples from AD patients [129]. However, in the hippocampal CA1 region and entorhinal cortex of the AD brain, BRCA1 protein was mislocalized to the cytoplasm and insoluble [123]. In line with the cytosolic mislocalization, the nuclear BRCA1 protein, but not other members of Defective DNA Repair (DDR) mechanisms, were found to be reduced in AD brains [130]. The cytoplasmic BRCA1 mislocalization may represent a consequence of TAU deposition, in line with the observation that brain regions without TAU pathology, namely the occipital lobe and the cerebellum, are free of cytoplasmic accumulation of BRCA1 despite decreased methylation of the coding gene. The insolubility of BRCA1 under the presence of aggregated TAU is proposed to be the reason for its dysfunction despite enhanced expression, contributing to the compromised genomic integrity of neurons and hence, disease pathophysiology [129]. BRCA1 was sequestered to TAU inclusions not only in AD brains, but also in brains of patients suffering from other tauopathies (namely PiD, PSP, CBD, FTDP17/FTLD-TAU) [130,131], strengthening the role for TAU in the disruption of DDR.

#### 2.2.4. Aβ-Associated Changes in DNA Methylation of Cell Cycle-Related Genes

In addition to compromised genomic integrity, dysregulated cell cycle control is an integral part of AD. While in a healthy neuron, abnormal cell cycle reentry leads to apoptosis, abnormal reentry in neurons of aged subjects with AD triggers a cycle of oxidative damage and mitogen production facilitating TAU hyperphosphorylation, Aβ deposition, and CI [132].

For genes promoting the activation of cell cycle reentry (i.e., via CDK5), hypomethylation was observed in AD or in AD disease paradigms [133]. Exposure of differentiated human neurons to Aβ results in DNA methylation abnormalities of cell-fate genes controlling neuronal differentiation and apoptosis, hinting at a downstream Aβ effect [133].

In this context, a recent study described a potential mechanism for DNA methylation-mediated Aβ overproduction, which then triggers Aβ driven hypomethylation of cell cycle-associated genes [134]. The same group (Li et al. (2019)) found that AD neurons display significant hypomethylation in the enhancer of the *DSCAML1* gene that targets *BACE1*. *BACE1* encodes the β-secretase, which cleaves APP thereby acting on Aβ production. Hence, the *DSCAML1* enhancer hypomethylation may activate *BACE1* transcription, putatively leading to an increased production of Aβ peptides, resulting in plaques typically preceding the spread of neurofibrillary tangles and neurodegeneration [135,136]. In agreement with this, changes of DNA methylation signatures in enhancer regulatory elements are frequently observed in AD brains [137,138]. Together, this indicates that epigenetic impairment of enhancer function is implicated in AD.

The studies described so far illustrate that changes in DNA methylation signatures can be elicited in response to pathophysiological processes induced in AD and/or tauopathies, making the role of DNA methylation in these diseases difficult to judge. Note that changes in neuronal activity can also modify the DNA methylation landscape [139], and that altered synaptic and neuronal function is a hallmark of AD and tauopathies.

The resulting changes in DNA methylation seem to contribute to the progressive neurodegeneration by transcriptional dysregulation, but the detailed implications require more investigation. Dissecting the relevance of DNA methylation for AD and other tauopathies is further complicated by newly arising scenarios of the biological relevance of DNA methylation. In addition to repressive promoter methylation, intragenic DNA methylation mediates alternative splicing and promoter choice. Moreover, apart from impeding transcription factor binding, certain DNA methylation patterns seem to create new motifs for transcription factors, for which increased methylation can also result in elevated expression [140]. Hence, the transcriptional consequences of certain changes in DNA methylation need to be dissected in much greater detail.

## 3. Epigenetic Treatment?—The Potential and Limitations of DNA Methylation-Based Therapy Approaches

As described above, hypomethylation of AD risk genes (such as *APP*, *PSEN1*, and *PSEN2*) was described to be associated with defects in learning and memory. An increase in methyl donor S-adenosyl-L-methionine (SAM) was reported to reduce APP and PSEN1 expression by promoter hypermethylation [141,142]. In line with this, elevated levels of vitamin B12, folate and other methionine sources in the diet improve methionine bioavailability and were shown to reverse elevated expressions of APP and PSEN1 [143,144,145].

In addition to driving hypermethylation, there is ongoing screening for DNMT inhibitors capable of modulating the methylation of AD or tauopathy risk genes. DNMT inhibitors such as azacitidine and decitabine have already been approved by the FDA for cancer treatment such as leukemia [146,147,148]. The use of DNA demethylating agents has also been used in some other neurodegenerative diseases, such as Friedreich’s ataxia [149], which however did not provide promising results in human cells.

**Figure 1:**
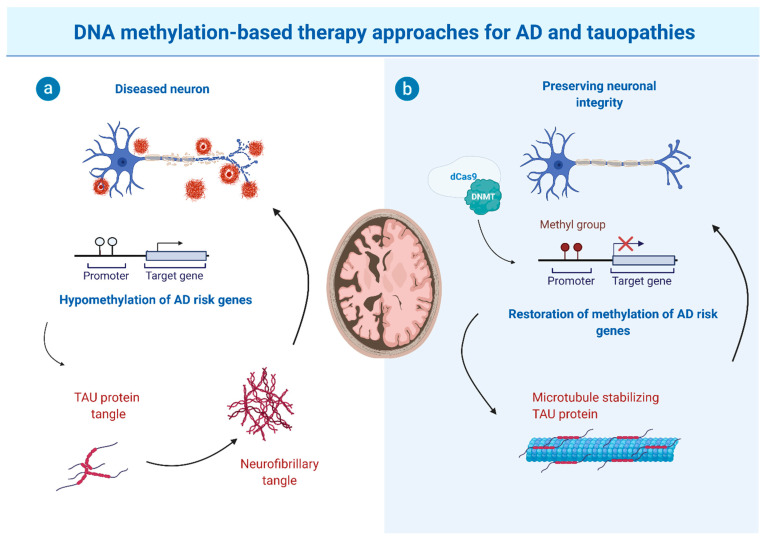
Putative potential of CRISPR/dCas9 editing-based therapeutic approaches for tauopathies that display impaired methylation patterns of selected genes/key regulator elements. (**a**) In disease paradigms, impaired DNA methylation (e.g., hypomethylation of risk genes associated with Alzheimer’s Disease (AD) and related/other tauopathies) results in increased TAU expression, decreased TAU clearance, or mislocalization, all of which lead to the accumulation of TAU and eventually to the formation of TAU protein aggregates. Neurons affected by this TAU pathology become dysfunctional and decay, eventually leading to impaired cognitive function and neurodegeneration. (**b**) CRISPR/dCas9 editing approaches may restore methylation patterns of AD and tauopathy risk genes, preventing abnormal production or modification of TAU protein and NFT formation, preserving the physiological function of TAU (i.a. microtubule stabilization) and preventing or partially reverse brain damage and disease progression. Possible genetic and non-genetic interventions could be (i) drug-induced modulation of methylation patterns, (ii) gene-replacement or RNAi-based gene therapy, or (iii) site/gene-specific modulation of methylation, e.g., as depicted, site-specific methylation via dCas9-directed DNMT targeting. This figure produced by using BioRender.com with a respective publication licence, provided by the Biology department of the RWTH Aachen University.

Finally, due to gene locus-specific changes in DNA methylation signatures, sequence-specific DNA demethylating agents, such as the oligonucleotide antisense inhibitor MG98 [150,151,152], seem promising for future therapeutic approaches to reduce DNA methylation site specifically. Moreover, the hypomethylation of particular genes was described to be implicated in AD and tauopathy pathomechanisms. Hence, locus-specific editing technologies are required for altering or restoring DNA methylation. This can be achieved by clustered regulatory interspaced short palindromic repeats (CRISPR)-deactivated Cas9 (dCas9)-based editing systems that have been described as a specific and efficient method capable of manipulating site-specific DNA methylation [153]. This, in combination with improvements in cell type-specific application and blood-brain-barrier overcoming strategies, would open the way for targeted epigenetic therapies (see Figure 1 for schematic depiction).

## 4. Altered DNA Methylation Signatures as Potential Biomarkers for AD/Tauopathies Disease and Disease Progression?

Disease-specific reliable biomarkers for difficult-to-diagnose diseases that might require early intervention (such as in current treatment approaches for AD and tauopathies) are essential for early diagnosis, monitoring disease progression, and eventually the response towards potential therapies. Currently used biomarkers, e.g., neurofilaments, are often unspecific and respond proportionally to the degree of axonal damage in a variety of neurological disorders, including inflammatory, neurodegenerative, traumatic, and cerebrovascular diseases, and is thus implicated in diseases reaching from stroke and TBI over ALS to prionopathies and many other sorts of neurological disorders. For some tauopathies, especially AD, biomarkers (mainly from the CNS) are established and aid in the diagnosis, e.g., lower Aβ and higher pTAU and tTAU levels. Yet, for most other tauopathies biomarkers are not established and are understudied. So, may DNA methylation signatures be useful to serve as biomarkers for AD and other tauopathies in blood cells, thereby complementing currently applied biomarkers?

In leukocytes, the intron 1 of the *TREM2* gene (triggering receptor expression on myeloid cells 2) displays reduced methylation, associated with elevated expression at the mRNA level in AD subjects [129,130]. Moreover, increased levels of peripheral *BDNF* promoter methylation was proposed to be an epigenetic biomarker indicating the transformation of MCI to AD [154]. Similarly, increased DNA methylation levels were detected in promoter regions of the *COASY* and *SPINT* genes in plasma samples of AD and MCI subjects compared to controls [155]. Methylation of the *PICALM* gene in blood cells was found to be related to the cognitive decline of AD patients [156]. Interestingly, global DNA methylation levels also were increased in peripheral blood (mononuclear cells) of LOAD patients, paralleled with an increase in the DNMT1 gene and protein expression, hinting towards global DNA methylation as a promising biomarker for AD, AD progression and AD conversion [157].

In sum, current studies indicate that monitoring global and site-specific DNA methylation in peripheral samples may be useful for individualized AD risk assessment. However, more detailed research and correlations are required that strengthen the use of DNA methylation as biomarkers for AD risk, diagnosis and progression, which might be expected in the near future.

## 5. Conclusions

Changes in DNA methylation seem to be critically implicated in causing and/or driving the progression of AD and tauopathies, for which epigenetic therapy strategies targeting DNMTs and DNA methylation are promising. However, there is still a long way to go. Firstly, locus-, cell-type and disease progression-specific changes have to be clearly dissected and correlated to the transcriptional output as well as the physiological consequences. Then, targeted strategies, such as being offered by (CRISPR)-deactivated Cas9 (dCas9)-based editing systems, have to be exploited and developed for site-specific manipulations of DNA methylation signatures. Finally, these manipulation systems have to be safely applicable to the brain and to specific neuronal subtypes at certain disease-progression stages. In addition to potential therapeutic targets, epigenetic signatures may also help to improve diagnosis of AD and tauopathies, for which epigenetic regulation likely will become an important tool in the treatment of these diseases.

## Figures and Tables

**Table 1:** Important examples of tauopathies with (epi)genetic etiologies or risk factors (see also [45,52,53,54]).

Disease Entity	Clinic Description/Overview	Etiology	Secondary to or Coexisting with
Familial FTLD-TAU due to coding mutations in *MAPT*	Very heterogenous group of aging associated tauopathies, which comprise i.a. formerly FTDP17(t) and patients diagnosed with PSP	Genetic: *MAPT*	-
Vacuolar tauopathy	FTLD-like syndrome due to defective TAU disaggregation	Genetic: *VCP* [55]	-
Other forms of FTLD-TAU (like) tauopathies	Heterogenous group of aging-associated tauopathies, like CBD, PiD, GGT, AGD, PART, ARTAG, most of which are further subclassified	Mostly sporadic, (epi)genetic causes unclear	-
Progressive supranuclear palsy (PSP)	Rare neurodegenerative disorder, but a common atypical Parkinson syndrome with cognitive, motor, behavior and language abnormalities, often diagnosed as AD	Epigenetic: Hypomethylation of *MAPT* [56] Genetic: *MAPT* Sporadic: GWAS with loci close to *MAPT*, *STX6*, *EIF2AK3*, *MOBP*, *DUSP*, *SLCO1A2*, *RUNX2*, i.a. [57]	-
PSP look-alike syndromes	Clinically similar to PSP, rare	Genetic: *LRRK2*, *DCTN1*, *BSN*	mostly unclear
Familial Alzheimer Disease	Age of Onset usually between 40 and 70 years, fast progression	Genetic: *APP*, *PSEN1*, *PSEN2*, up to ~75 risk modifying genes	Amyloid-pathology
Familial Parkinson Disease	Various group of familial Parkinson Syndromes	Genetic: *SNCA*, *PRKN*, *LRRK2*, other	alpha-Synuclein deposits
Familial FTLD-ALS Syndromes	Syndromes with manifestations ranging from pure ALS to pure FTLD or overlapping phenotypes	Genetic: *GRN*, *C9ORF72*, *TARDBP*, other	Deposits of dipeptide repeats, RNA inclusions, TDP-43
Hereditary cerebral amyloid angiopathy	Familial forms of dementia (fam. British and fam. Danish dementia)	Genetic: *ITM2B*	Amyloid-pathology
Niemann Pick Disease Type C	Lysosomal storage disease with hepatosplenomegaly, progressive dementia, and premature death ranging from infancy to late adulthood	Genetic: *NPC1*, *NPC2*	Cholesterol accumulations
Kufs Disease	A neurodegenerative lysosomal storage disease/neuronal ceroid lipofuscinosis	Genetic: *CLN6 (PPT1*, *DNAJC5*, *CTSF)*	Lipofuscin accumulations
Christianson Syndrome	X-linked mental retardation syndrome with microcephaly, muscle hypotonia, movement disorder, and epilepsy	Genetic: *SLC9A6*	-/unclear
Mental Retardation, X-linked, syndromic, Hedera type	X-linked mental retardation syndrome with global developmental delay, parkinsonism, spasticity, and progressive neurodegeneration	Genetic: *ATP6AP2*	SQSTM1 depositions
Myotonic Dystrophy Type 1 & 2	Most common forms of muscular dystrophy characterized by muscle weakness, progressive muscle loss, and may include cataracts, diabetes, and dementia at late stages	Genetic: *DMPK*, *CNBP*	RNA nuclear inclusions
(Infantile) Sialic Acid Storage Disease	NDD with lysosomal dysfunction presenting in infancy in its severe form or in adulthood with progressive brain atrophy	Genetic: *SLC17A5*	-/unclear
PKAN	NDD with brain iron accumulation	Genetic: *PANK2*	Iron depositions
